# Socioeconomic factors impact the risk of HIV acquisition in the township population of South Africa: A Bayesian analysis

**DOI:** 10.1371/journal.pgph.0001502

**Published:** 2023-01-26

**Authors:** Cindy Leung Soo, Nitika Pant Pai, Susan J. Bartlett, Aliasgar Esmail, Keertan Dheda, Sahir Bhatnagar

**Affiliations:** 1 Department of Epidemiology, Biostatistics, and Occupational Health, McGill University, Montreal, Canada; 2 Department of Medicine, McGill University, Montreal, Canada; 3 Research Institute of the McGill University Health Centre, Montreal, Canada; 4 Centre for Lung Infection and Immunity, Division of Pulmonology, UCT Lung Institute and Department of Medicine, University of Cape Town, Cape Town, South Africa; The Aurum Institute, SOUTH AFRICA

## Abstract

With a prevalence almost twice as high as the national average, people living in South African townships are particularly impacted by the HIV epidemic. Yet, it remains unclear how socioeconomic factors impact the risk of HIV infection within township populations. Our objective was to estimate the extent to which socioeconomic factors (dwelling situation, education, employment status, and monthly income) explain the risk of HIV in South African township populations, after controlling for behavioural and individual risk factors. Using Bayesian logistic regression, we analysed secondary data from a quasi-randomised trial which recruited participants (N = 3095) from townships located across three subdistricts of Cape Town. We controlled for individual factors (age, sex, marital status, testing history, HIV exposure, comorbidities, and tuberculosis infection) and behavioural factors (unprotected sex, sex with multiple partners, with sex workers, with a partner living with HIV, under the influence of alcohol or drugs), and accounted for the uncertainty due to missing data through multiple imputation. We found that residing in informal dwellings and not having post-secondary education increased the odds of HIV (aOR, 89% CrI: 1.34, 1.07–1.68 and 1.82, 1.29–2.61, respectively), after controlling for subdistrict of residence, individual, and behavioural factors. Additionally, our results suggest different pathways for how socioeconomic status (SES) affect HIV infection in males and female participants: while socioeconomic factors associated with lower SES seem to be associated with a decreased likelihood of having recently sough HIV testing among male participants, they are associated with increased sexual risk taking which, among female participants, increase the risk of HIV. Our analyses demonstrate that social determinants of health are at the root of the HIV epidemic and affect the risk of HIV in multiple ways. These findings stress the need for the deployment of programs that specifically address social determinants of health.

## Introduction

With an estimated 19.1% of the population living with HIV, South Africa has one of the highest HIV rates in the world [1]. Within the country, the burden of HIV is unequally distributed across sociodemographic factors as well as geographically [2]. South African townships are urban settlements located within subdistricts of metropolitan areas which were originally designated during apartheid for non-whites only [3]. People living in townships are particularly impacted by the HIV epidemic, having a prevalence that is nearly twice as high as the national average [4]. Despite the availability of testing interventions and pre-exposure prophylaxis, incident HIV infections are being reported from South Africa. At the recent AIDS Conference in Montreal, the need to explore predictors of HIV infection that perpetuate inequity and predispose individuals to HIV was clearly highlighted. In these analyses, we set out to characterize how socioeconomic factors impact HIV in township populations of South Africa to guide informed policy decisions.

In Cape Town, 17% of households live in informal dwellings according to the 2021 General Household Survey [5]. Additionally, an estimated 23% of households in Cape Town experience food instability and 21% do not have a source of piped drinking water in their dwelling [5]. People of lower socioeconomic status (SES) are particularly vulnerable to stressors such as poverty and food insecurity which has been suggested to increase sexual risk taking and the risk of acquiring HIV over time [6].

Prevention strategies for HIV have been dominated by interventions aimed at influencing knowledge, attitudes, and behaviours [7]. While behavioural factors play a major role in the transmission of HIV, they poorly explain health inequities [8], which are health disparities that are unnecessary, avoidable, unfair, and unjust [9]. By contrast, how socioeconomic factors such as education, employment status, dwelling situation, and income impact HIV infection can not only inform public health policy implementation, but also social policies aimed at mitigating health inequities.

Previous studies have reported on the protective effect of education and a stable dwelling situation on HIV in South Africa, but there has been no consistent association between employment status and HIV acquisition [10, 11]. It also remains unclear how income or wealth affects the risk of HIV. While many have suggested that socioeconomic stressors could compel individuals of lower SES to adopt riskier sexual behaviours including transactional sex [6, 12, 13], more evidence is required showing how socio-economic indicators affect people living with HIV in South Africa when controlling for behavioural and demographic factors.

The objective of this study was to estimate the extent to which socioeconomic factors (education level, dwelling situation, employment status, and monthly income) explain the risk of HIV in South African township populations located within subdistricts of Cape Town, while controlling for individual and behavioural factors. In exploratory analyses, we also aimed to estimate the effect of socioeconomic factors on certain sexual behaviours and the effect of individual, behavioural and socioeconomic factors on HIV testing history.

## Methods

### Design and setting

Using data from a quasi-randomised trial in Cape Town, South Africa [14], we conducted a secondary data analysis. The trial was conducted between January 2017 and June 2018 and aimed to evaluate HIVSmart!, a digital self-testing programme for HIV with an oral self-test, on impact outcomes such as linkage to care, detection of new HIV infections, and increased referrals to self-test [14]. HIVSmart! is an Android/iOS app and web-based digital HIV self-testing programme, with a secure dashboard. It also has a Health Insurance Portability and Accountability Act (HIPAA)-compliant cloud based encryption as well as a peer worker-based 24/7 counselling service [15–17].

The study was conducted out of six community clinics distributed within subdistricts of Cape Town. Participants were recruited from three subdistricts of Cape Town (denoted here as subdistrict A, B, and C) and were offered either HIV self-testing or conventional testing. New infections were ascertained using an oral HIV self-test (OraQuick advance HIV-1/2, OraSure Technologies Inc, USA) in the self-testing arm and blood-based rapid test in the conventional testing arm. HIV test results were confirmed using a blood based p24 antigen rapid test and laboratory HIVRNA tests. For the analyses, the laboratory HIVRNA test results were used to define incident HIV infection. Further details of the primary study processes are published elsewhere [14].

### Measures

Individual factors included age, sex (female/male), marital status (married/not married), HIV testing history (tested in the last six months / not tested in the last six months), HIV exposure in the last six months (yes/no/abstain) as well as comorbidities (i.e., tuberculosis (TB), other lung infection, diabetes, hypertension, and asthma).

Behavioural risk factors included history of drug injection, and sexual risk factors such as unprotected sex, sex with sex workers, sex with multiple partners, sex with HIV infected individuals, and sex under the influence of alcohol and/or drugs (yes/no/abstain).

Socioeconomic variables assessed included post-secondary education (yes/no), type of dwelling (hostel or informal / house), employment status (working part-time or full-time / unemployed or retired), and monthly income (less than 3000 rand / 3000–6000 rand / 6001–9000 rand / over 9000 rand).

Measured variables were selected based on knowledge of HIV epidemiology during the design of the HIVSmart! trial.

### Statistical analysis

Variables were summarised using mean, standard deviation (SD), median, interquartile range and range for continuous variables, with a Kruskal-Wallis rank sum test p-value comparing the subdistricts. Categorical variables were summarised by count and frequency with a Pearson’s Chi-squared test p-value comparing the subdistricts.

We deployed Bayesian statistics which offers two key advantages compared to frequentist approaches: it allows the incorporation of prior information and provides more intuitive and meaningful inferences. We used Bayesian logistic regression to estimate the effect of subdistrict of residence, individual, socioeconomic, and behavioural factors on the risk of acquiring HIV. We report the posterior medians as well as the 89% credible intervals (CrI) of the adjusted odds ratios. We chose to report the 89% CrI rather than the 95% CrI since the 95% CrI can be unstable when the effective sample sizes are low (< 10,000) [18]. However, both the 89% and 95% credible intervals are plotted in the figures.

Priors for age and sex were informed by a study by Bärnighausen et al. [19]. The prior for age was set as normal prior with a mean of log(0.9954) and a standard deviation of 1. The prior for sex was set as a normal prior with a mean of log(1.8314) and an SD of 1. The prior for the beta coefficient of all other variables was specified as a normal prior with a mean of 0 and an SD of 1. The goodness-of-fit of all models were evaluated using the Cressie-van Houwelingen test and by visualising the bootstrap resampling calibration curves [20].

The missing data was assumed to be missing at random and was imputed using multiple imputation by chained equations with five imputed datasets. Results were pooled from the posterior draws of the five submodels to account for the additional uncertainty caused by the imputation procedure [16, 17].

Analyses were performed in R version 4.0.2 [21]. Multiple imputation was performed using the *mice* package [22] and Bayesian modelling was performed using the *brms* package [23]. We used the *rms* package to evaluate the goodness-of-fit of our models [24], the *tidyverse* package [25] for data wrangling and visualisation and the *arsenal* package [26] to create summary tables.

### Ethics approval

The trial was approved by the Institutional Review Boards of the Research Institute of the McGill University Health Centre (RI MUHC) and the University of Cape Town. Ethics approvals for secondary data analyses were obtained from both the RI MUHC and the University of Cape Town.

## Results

### Demographics

In total, 3095 participants were recruited [14] of which 212 (7%) were missing at least one variable and 81 (3%) were missing more than five variables. 893 (29%) participants were from subdistrict A, 1023 (33%) from subdistrict B, and 1112 (36%) from subdistrict C of Cape Town (Table 1). 64 (2%) participants did not specify their subdistrict of residence. Participants were on average 29 years old, predominantly female (70%), and unmarried (71%). Close to half (48%) had tested for HIV in the past six months but were undiagnosed and not on treatment at the time of testing.

**Table 1:** Baseline characteristics of participants in subdistricts of Cape Town.

	Subdistrict A (N = 893)	Subdistrict B (N = 1023)	Subdistrict C (N = 1112)	Overall (N = 3095)	p-value^§^
**Missing subdistrict**				64	
**Individual Factors**					
**Age**					
Missing	0	0	1	1	
Mean (SD)	28 (8)	29 (8)	29 (9)	29 (9)	< 0.001
Median (IQR)	26 (22–32)	27 (22–33)	27 (22–34)	27 (22–33)	
Range	17–67	17–67	17–76	17–76	
**Sex**					
Missing	0	1	1	2	
Male	264 (29.6%)	300 (29.4%)	336 (30.2%)	919 (29.7%)	0.896
Female	629 (70.4%)	722 (70.6%)	775 (69.8%)	2174 (70.3%)	
**Marital status**					
Missing	11	29	36	81	
Unmarried	606 (68.7%)	678 (68.2%)	828 (77.0%)	2153 (71.4%)	< 0.001
**Previous HIV test**					
Missing	0	1	0	64	
Has not tested in the past 6 months	464 (52.0%)	611 (59.8%)	494 (44.4%)	1573 (51.9%)	< 0.001
**In the past 6 months, have you been exposed to HIV?**					
Missing	0	1	0	64	
Yes	43 (4.8%)	63 (6.2%)	73 (6.6%)	179 (5.9%)	0.529
Abstain	13 (1.5%)	17 (1.7%)	19 (1.7%)	49 (1.6%)	
**Have you ever been diagnosed with tuberculosis?**					
Yes	68 (7.6%)	70 (6.8%)	106 (9.5%)	247 (8.0%)	0.063
**Have you ever been diagnosed with any of these illnesses:** **lung infection, diabetes, hypertension, asthma?**					
Yes	77 (8.6%)	136 (13.3%)	85 (7.6%)	305 (9.9%)	< 0.001
**Behavioural Factors**					
**Are you sexually active?**					
Missing	1	3	7	74	
Yes	811 (90.9%)	906 (88.8%)	983 (89.0%)	2704 (89.5%)	0.142
Abstain	24 (2.7%)	22 (2.2%)	36 (3.3%)	82 (2.7%)	
**In the past 6 months, have you injected drugs (excluding medicine)?**					
Missing	0	1	0	64	
Yes	23 (2.6%)	34 (3.3%)	43 (3.9%)	100 (3.3%)	0.167
Abstain	3 (0.3%)	10 (1.0%)	12 (1.1%)	25 (0.8%)	
**In the past 6 months, I have had sex… (select all that applies)**					
Missing	5	5	8	81	
Without a condom	566 (63.7%)	716 (70.3%)	811 (73.5%)	2097 (69.6%)	< 0.001
With multiple partners	83 (9.3%)	113 (11.1%)	107 (9.7%)	303 (10.1%)	0.391
With a commercial sex worker	5 (0.6%)	6 (0.6%)	13 (1.2%)	24 (0.8%)	0.203
With an HIV infected partner	15 (1.7%)	28 (2.8%)	34 (3.1%)	77 (2.6%)	0.132
Under the influence of alcohol	84 (9.5%)	129 (12.7%)	103 (9.3%)	317 (10.5%)	0.021
Under the influence of drugs (e.g. marijuana, cocaine, heroin, etc.)	12 (1.4%)	29 (2.8%)	23 (2.1%)	64 (2.1%)	0.077
Abstain	13 (1.5%)	23 (2.3%)	23 (2.1%)	59 (2.0%)	0.428
**Socioeconomic Factors**					
**What is your highest level of education?**					
Graduate, undergraduate or college	173 (19.4%)	207 (20.2%)	199 (17.9%)	593 (19.2%)	0.38
High school, primary school or no schooling	720 (80.6%)	816 (79.8%)	913 (82.1%)	2502 (80.8%)	
**What sort of dwelling do you live in?**					
Formal house or other	489 (54.8%)	457 (44.7%)	584 (52.5%)	1568 (50.7%)	< 0.001
Hostel or informal dwelling	404 (45.2%)	566 (55.3%)	528 (47.5%)	1527 (49.3%)	
**What is your work situation?**					
Employed (part-time or full-time)	349 (39.1%)	468 (45.7%)	470 (42.3%)	1288 (41.6%)	0.013
Not employed or retired	544 (60.9%)	555 (54.3%)	642 (57.7%)	1807 (58.4%)	
**What is your monthly income?**					
Missing	0	0	3	66	
>9000 R	20 (2.2%)	32 (3.1%)	56 (5.0%)	108 (3.6%)	< 0.001
6001–9000 R	28 (3.1%)	50 (4.9%)	29 (2.6%)	107 (3.5%)	
3000–6000 R	135 (15.1%)	171 (16.7%)	150 (13.5%)	457 (15.1%)	
<3000 R	710 (79.5%)	770 (75.3%)	874 (78.8%)	2357 (77.8%)	

^§^Missing information was not included in the analysis.

### Differences across subdistricts

Participants from subdistrict A were on average younger while participants from subdistrict C were slightly older (Table 1). As compared with subdistrict C, a smaller proportion of participants from subdistrict A and subdistrict B were unmarried (% unmarried, 77.0%, 68.7%, 68.2%, respectively). Participants from subdistrict B were less likely to have tested in the past six months.

Other factors which differed across subdistricts are frequency of comorbidities, whether engaging in unprotected sex or sex under the influence of alcohol, dwelling situation, employment situation, and monthly income (Table 1).

### The odds of HIV infection differed across subdistricts of Cape Town, South Africa

**Fig 1:**
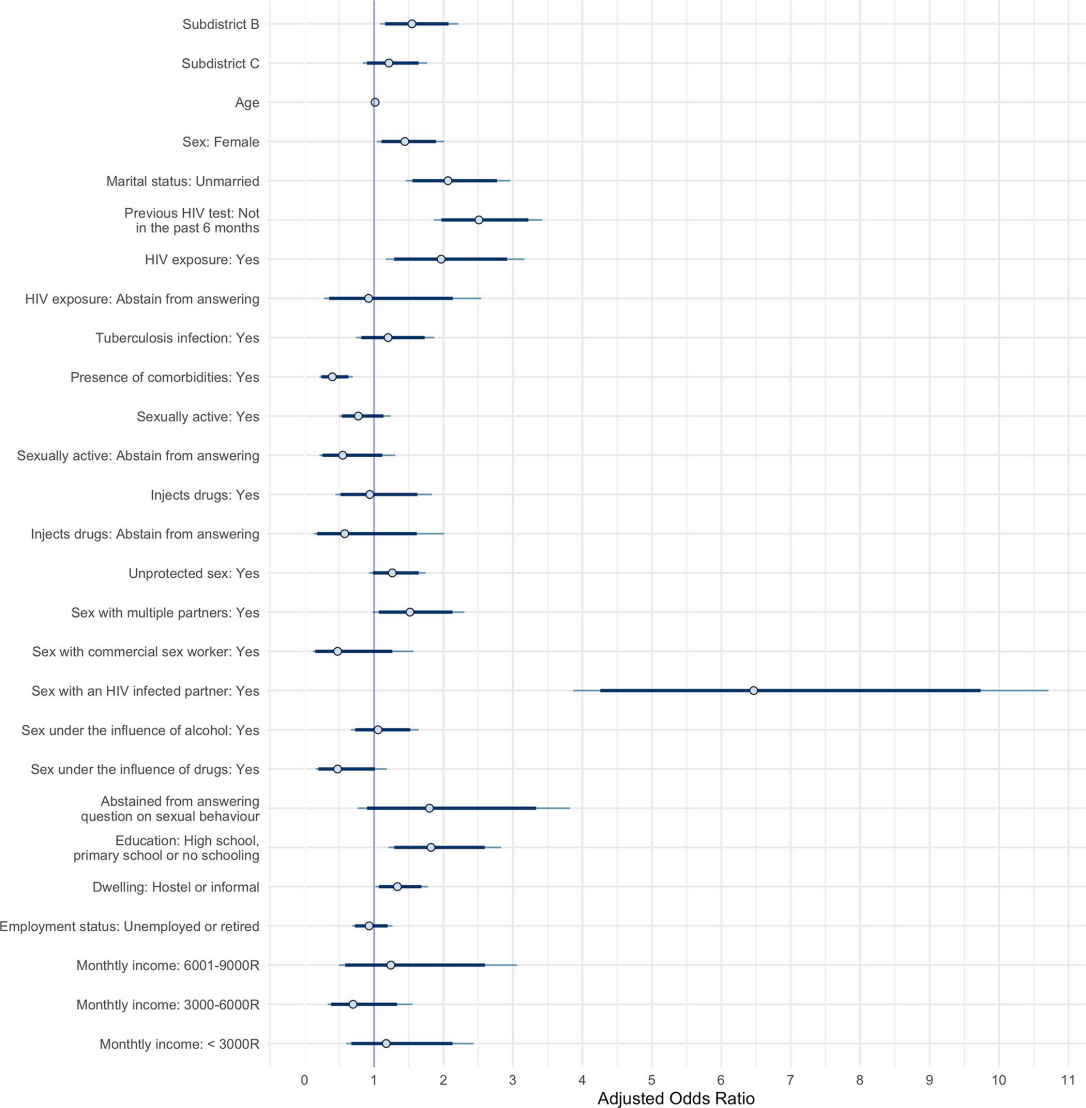
Bayesian logistic regression model posterior intervals of adjusted odds ratios of HIV infection. Points represent the posterior medians, thick segments represent the 89% credible intervals and thin segments represent the 95% intervals of the adjusted odds ratios.

The goodness-of-fit of the logistic regression models predicting HIV status were deemed appropriate (p > 0.05) and the calibration curves were found to be satisfactory (Fig A in S3 Text). We found that those residing in subdistrict B and C were at increased odds of being HIV positive compared to residents of subdistrict A in unadjusted analyses (Table A in S1 Text). This difference persisted even after controlling for individual, behavioural, and socioeconomic factors, although the null effect was within the 89% probability bounds of the odds ratio for subdistrict C (aOR, 89% CrI: 1.54, 1.16–2.07 and 1.21, 0.90–1.64, respectively) (Fig 1).

### Less stable housing situation and lower education level increased the odds of HIV infection

In adjusted analyses, individuals living in hostels or informal dwellings were at greater odds of being HIV positive (aOR, 89% CrI: 1.34, 1.07–1.68) relative to those living in a house. In addition, individuals with lower education also had increased odds of HIV infection (aOR, 89% CrI: 1.82, 1.29–2.61) relative to those having achieved post-secondary education or higher in our adjusted analysis (Fig 1).

### Engaging in unprotected sex, sex with multiple partners, or sex with an HIV infected partner increased the odds of HIV infection among female participants

Having had sex with a partner living with HIV was associated with the highest adjusted odds ratio of infection (aOR, 89% CrI: 6.43, 4.22–9.74), after controlling for all other factors. Our sex-stratified analyses indicated that while female participants who engaged in unprotected sex or sex with multiple partners were more likely to be affected by HIV (aOR, 89% CrI: 1.37, 1.01–1.89 and 2.09, 1.31–3.27, respectively), the odds of HIV for male participants were not impacted by whether or not they had engaged in condomless sex or sex with multiple partners (Fig A in S2 Text). Female participants who chose not to disclose sexual behaviours were also at increased odds of HIV (aOR, 89% CrI: 2.18, 1.00–4.59) (Fig A in S2 Text). By contrast, those who reported having had sex under the influence of drugs had lower odds of being HIV positive in adjusted analyses (aOR, 89% CrI: 0.47, 0.20–1.02), although the 89% probability bound incuded the null effect (Fig 1). Sex-stratified analyses revealed that this effect was mostly predominant in male participants (aOR, 89% CrI: 0.34, 0.12–0.88) (Fig A in S2 Text).

### Sex, marital status, having pre-existing conditions, testing history, and previous HIV exposure affected the odds of new HIV infection

Female participants were at increased odds of being HIV positive compared to male participants (aOR, 89% CrI: 1.44, 1.11–1.89), after controlling for all other individual, behavioural, and socioeconomic factors as well as subdistrict of residence. In our adjusted analyses, those who were unmarried or had not tested in the previous six months were more likely to be affected with HIV (aOR, 89% CrI: 2.07, 1.56–2.76 and 2.51, 1.97–3.23, respectively). Notably, male participants who had not tested for HIV in the previous 6 months were over three times more likely to be affected by HIV (Fig A in S2 Text). The effect of tuberculosis (TB) infection differed depending on sex; women infected with TB were more likely to be living with HIV (aOR, 89% CrI: 1.60, 1.04–2.54) while TB infection did not appear to significantly impact HIV infection among male participants (Fig A in S2 Text). Those with other comorbidities had lower odds of testing positive for HIV (aOR, 89% CrI: 0.39, 0.24–0.63). Finally, those who indicated having been exposed to HIV in the past six months were at increased odds of HIV infection (Fig 1), an effect which appear to be stronger among male participants (Fig A in S2 Text).

### Socioeconomic factors impacted the odds of engaging in sexual behaviours associated with HIV infection

In exploratory analyses, we investigated whether socioeconomic factors impacted sexual behavioural factors associated with HIV infection, specifically engaging in unprotected sex, sex with multiple people, or sex with a person living with HIV, after controlling for age and sex. We found the goodness-of-fit as well as the resampling calibration plots of the models predicting unprotected sex, sex with multiple people, or sex with a person living with HIV to be satisfactory (p > 0.05) (Figs B-D in S3 Text).

**Fig 2:**
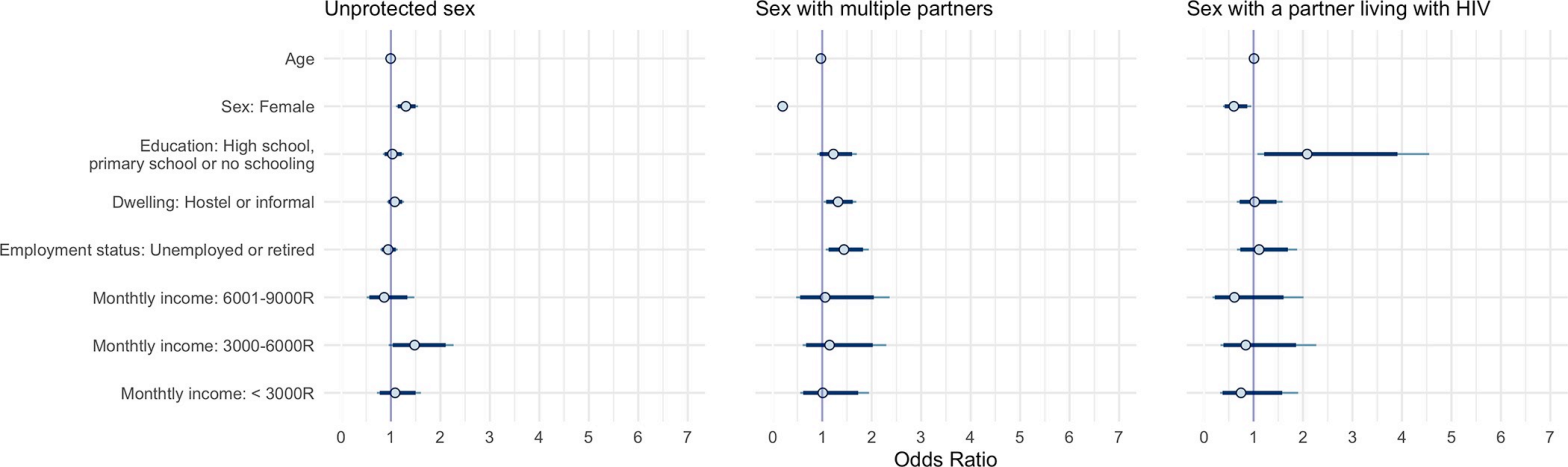
Bayesian logistic regression model posterior intervals of adjusted odds ratios of the impact of socioeconomic factors on select behavioural factors. Points represent the posterior medians, thick segments represent the 89% credible intervals and thin segments represent the 95% intervals of the adjusted odds ratios.

According to our analyses, individuals in the 3000 – 6000R per month income level were at increased odds of engaging in unprotect sex (aOR, 89% CrI: 1.48, 1.04–2.11) compared to those in the highest income level (> 9000R per month), after adjusting for age, sex, and other socioeconomic factors (Fig 2). Sex-stratified analyses revealed that this effect was specific to female participants (aOR, 89% CrI: 1.91, 1.24–2.94) (Fig B in S2 Text). Female participants in this income strata were also less likely to have sex with multiple partners (aOR, 89% CrI: 0.32, 0.13–0.77) whereas male participants with similar income were more likely to have multiple sexual partners (aOR, 89% CrI: 2.05, 1.09–3.94), compared to participants in the highest income level (Fig B in S2 Text). Our adjusted analyses also revealed increased odds of engaging in sex with multiple partners for participants with a less stable housing situation (aOR, 89% CrI: 1.32, 1.08–1.62) and those who are not currently employed (aOR, 89% CrI: 1.44, 1.13–1.83) (Fig 2). The effect of less stable dwelling situation on having multiple sexual partners appears to be stronger among male participants (aOR, 89% CrI: 1.45, 1.11–1.89) (Fig B in S2 Text). Finally, individuals without post-secondary education were over two times more likely to engage in sex with a partner living with HIV in adjusted analyses (Fig 2). This effect was particularly strong among female participants (aOR, 89% CrI: 2.53, 1.22–5.60) (Fig B in S2 Text).

### Socioeconomic factors appear to impact the likelihood of having been tested for HIV in the past six months among male participants

Given the strong association we observed between HIV infection and testing history we examined, in exploratory analyses, the relationship between individual, behavioural, and socioeconomic factors and having sought HIV testing in the past six months. The goodness-of-fit and the resampling calibration curves of the models predicting an HIV test in the previous six months were deemed appropriate (p > 0.05) (Fig E in S3 Text).

In unadjusted analyses, individuals residing in subdistrict B were less likely to have been tested in the past six months compared to residents of subdistrict A (OR, 89% CrI: 0.74, 0.64–0.86) (Table B in S1 Text). By contrast, those from subdistrict C of Cape Town were more likely to have sought HIV testing the past six months relative to subdistrict A residents, although the null effect was within the 89% probability bounds of the estimated odds ratio (OR, 89% CrI: 1.36, 1.18–1.56) (Table B in S1 Text).

**Fig 3:**
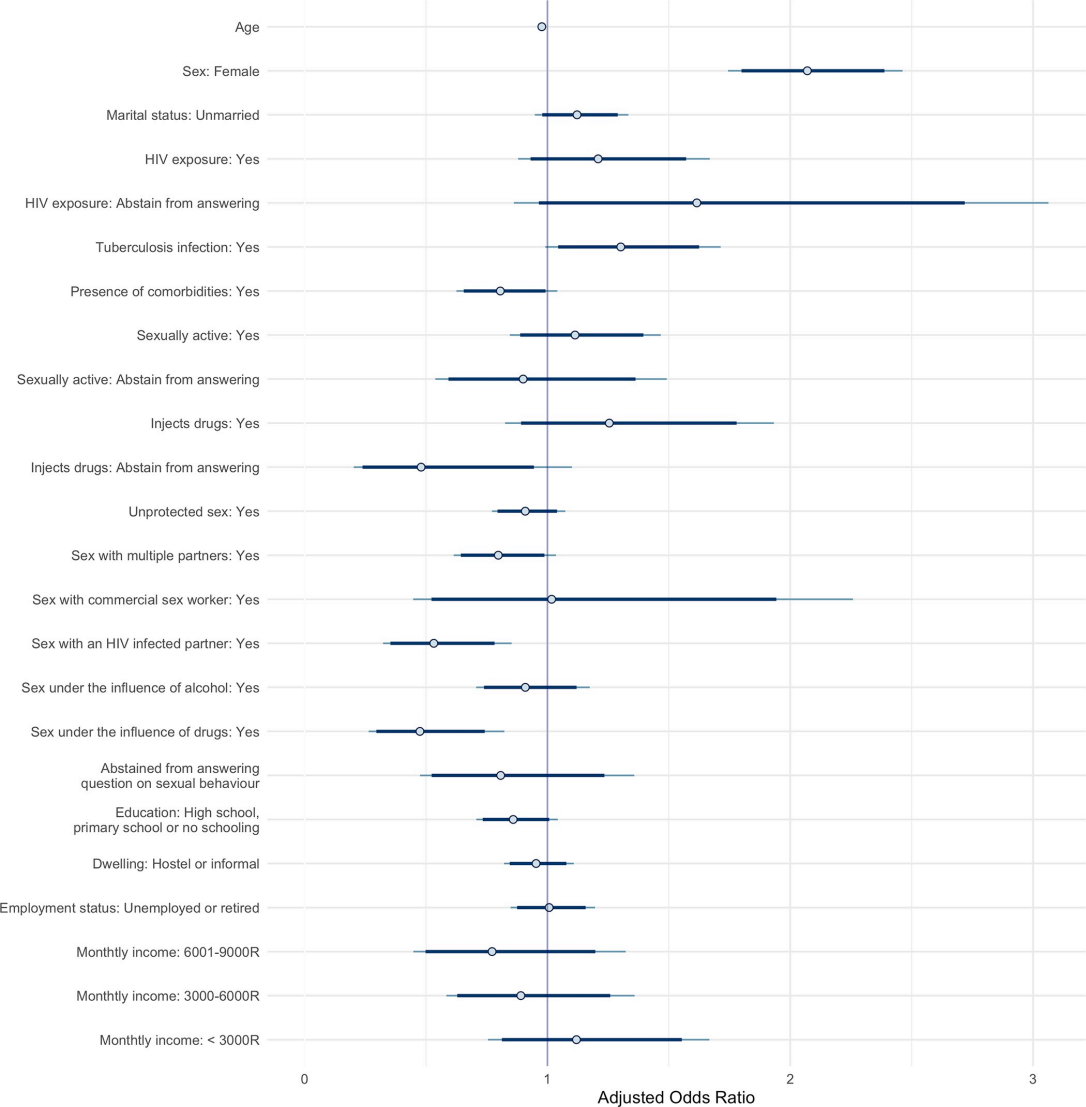
Bayesian logistic regression model posterior intervals of adjusted odds ratios for having tested in the past six months. Points represent the posterior medians, thick segments represent the 89% credible intervals and thin segments represent the 95% intervals of the adjusted odds ratios.

Each additional year of age decreased the odds of having been tested for HIV in the past six months by 2% (Fig 3). Further, we found that women were approximately twice as likely as men to have been tested for HIV in the past six months. Female participants living with tuberculosis were also more likely to have recently sought HIV testing (Fig C in S2 Text).

On the other hand, individuals with comorbidities or who were married were less likely to have recently been tested for HIV. Behavioural factors associated with lower odds of testing in the past six months include having engaged in unprotected sex, having had sex with multiple partners, an HIV infected person or under the influence of drugs (Fig 3).

Finally, male participants without post-secondary education and living in less stable dwelling situations were slightly less likely (aOR, 89% CrI: 0.76, 0.57–1.03 and 0.81, 0.64–1.02, respectively) to have sought testing in the past six months, although the null effect was within the 89% probability bounds for the estimated odds ratio (Fig C in S2 Text).

## Discussion

In these analyses, we have demonstrated that socioeconomic factors such as unstable housing and lower education level impact the odds of HIV infection, even after controlling for individual and behavioural factors. Our results suggest different pathways for how these social determinants of health impact HIV risk in female and male participants. Female participants having lower education level appeared to have increased odds of engaging in certain sexual behaviours which were associated with increased HIV risk. Yet, none of the socioeconomic indicators appeared to significantly alter the likelihood of having sought HIV testing in the previous 6 months among female participants. By contrast, male participants living in less stable housing situations or having lower education levels appeared less likely to have recently tested for HIV, a factor which was strongly associated with an increased likelihood of HIV infection. Most behavioural factors, on the other hand, did not appear to impact the odds of HIV among male participants. Overall, our findings indicate that social determinants of health can affect HIV infection in different ways but nevertheless remain key enablers of the HIV epidemic, irrespective of sex.

Other studies have previously reported on the impact of social determinants on the risk of HIV in South Africa [7, 10, 19, 27]. One study found that those living in “urban informal areas” had over twice the odds of HIV prevalence compared to those in “urban formal areas” [28], consistent with our own findings that less stable housing increased the odds of being HIV positive even after controlling for individual and behavioural factors. Additionally, in line with what our adjusted analyses showed, several studies have previously reported that educational attainment reduces the risk of HIV infection [10, 19, 27].

It has been suggested that formal education can empower individuals to engage with prevention initiatives and adopt risk reducing behaviours more effectively [19]. Our finding appear to corroborate this mechanism, particularly among female participants. Indeed, the results of our exploratory analyses indicate that the odds of engaging in sex with multiple partners or with a partner living with HIV were higher for female participants with lower levels of education. On the other hand, educational attainment was not significantly associated with these behavioural risk factors in male participants, nor was it associated with the odds of engaging in unprotected sex. Furthermore, our analyses showed that engaging in unprotected sex and sex with multiple partners, increased the odds of HIV infection among female participants, in line with what had previously been described [29], but not among male participants.

Our results suggest that socioeconomic factors impact the HIV risk of male participants differently. Indeed, it would seem from our analyses that male participants living in unstable dwelling or having lower education were less likely to have recently tested for HIV, although the 89% probability bounds for both factors included the null effect. This finding, which in our case was specific to male participants, is consistent with previous studies that have reported an association between lower socioeconomic status and a decreased likelihood of HIV testing [28, 30, 31]. It also echoes concerns that men remain an under-tested population in South Africa when it comes to HIV [32, 33]. Repeat testing is an important strategy to address the HIV epidemic as it often permits early detection of the virus and early initiation of antiretroviral therapy (ART) [34]. Consequently, repeat testers have been shown to have a higher CD4 T cell counts when an HIV infection is diagnosed, an earlier start of ART as well as a reduced mortality. Early detection of the virus through repeat testing may also reduce onward transmission of the virus through engaging more people in ART which lowers viral load and infectivity.

While we found no significant association between employment status and HIV infection, consistent with previous studies [10, 11], our exploratory analyses found that not being employed increased the odds of engaging in sex with multiple partners. Having multiple sexual partners was associated with a greater risk of HIV infection among female participants suggesting that it is a potential mediating factor between employment status and HIV infection for women. Indeed, several socioeconomic stressors stemming from or intersecting with unemployment, including poverty and food insecurity, can constrain individuals to engage in risky health behaviours such as unprotected transactional sex [6] making these individuals particularly vulnerable to HIV and further deepening the impact of health inequities.

Finally, consistent with previous reports, women [28, 29, 35], unmarried individuals [29, 35], and individuals who had not tested for HIV in the previous six months were also at increased odds of HIV infection in our adjusted analyses.

HIV prevention strategies in South Africa and globally have very much been focused on addressing individual risk of HIV through encouraging the use of condoms, promoting sexual education, offering voluntary medical male circumcision, and targeting pre-exposure prophylaxis as well as HIV testing (self-testing, community and facility based testing) to those deemed at highest risk [36, 37]. However, our results indicate that individual and behavioural factors do not fully explain the risk of acquiring HIV infection; socioeconomic factors also impact the odds of HIV in subdistricts of South Africa. Indeed, while socioeconomic factors may impact males and females’ risk of HIV in different ways, they remain at the root of the HIV epidemic in South Africa.

The contribution of socioeconomic factors in the HIV epidemic has been overshadowed by the emphasis on individual and behavioural factors in prevention initiatives, and a greater focus on addressing social determinants of health is warranted. On the topic of public health policies, Geoffrey Rose famously argued that population strategies aimed at targeting population determinants of disease should be favoured over high-risk strategies as they address the root causes of incidence of disease, and they are behaviourally appropriate in that they do not discriminate across individual susceptibility [38]. The high-risk strategy, focused on key populations and behavioural risk factors, has been a dominant approach for HIV prevention programmes, but they poorly address the underlying social determinants of health. On the other hand, population strategies addressing socioeconomic factors by promoting more stable housing and higher educational attainment can have a positive impact on the incidence of HIV by shifting the disease curve of the entire population. As Rose points out, this approach is not only radical in its attempt to alter the underlying causes of disease but also non-discriminatory [38] which is particularly welcomed in the context of HIV, where discrimination and stigma towards people living with HIV have been important impediments to the progress towards the elimination of the disease [37].

### Limitations

Several limitations are associated with our present study. Since we used secondary data collected from subdistricts of Cape Town, the results of our analyses may not generalize to populations of different contexts. Further, the potential for social desirably bias cannot be ruled out, although participants had the option to abstain from answering questions to mitigate this. The presence of residual confounding is also plausible given the broad strata within our categorical variables. Some variables had few observations per strata, possibly leading to some imprecision in the reported credible intervals [16, 17]. Finally, we recognise that the secondary data which was available to us may not have been well suited to detect differences in HIV infection across factors which were distributed relatively homogenously in our study population. Replicating these analyses within a population with greater heterogeneity in socioeconomic factors could help shed a better light on the impact of SES on HIV infection.

## Conclusions

Through these analyses, we demonstrated that socioeconomic factors such as less stable housing, lower education level, as well as subdistrict of residence impact the odds of HIV infection even after controlling for individual and behavioural factors. While HIV prevention strategies have typically emphasised personal responsibility in health, our analyses show that individual and behavioural factors alone cannot fully explain HIV infection. Additionally, we have shown that socioeconomic factors impact HIV risk in male and female participants in different ways. Indeed, factors associated with lower SES appear to be associated with a reduced likelihood of having recently sough HIV testing specifically among male participants. On the other hand, these socioeconomic factors were associated with increased sexual risk taking which, among female participants, increased the risk of HIV. Altogether, our findings stress the need to diversify HIV prevention strategies and implement social policies which aim to improve education, living conditions as well as possibly other intersecting socioeconomic factors including employment and income stability in the aim of tackling health inequities in the HIV epidemic.

## Supporting information

S1 Text Posterior medians, 89% and 95% credible intervals (CrI) of unadjusted analyses. (PDF)

S2 Text Bayesian logistic regression models stratified by sex. (PDF)

S3 Text Resampling model calibration. (PDF)

S1 Data Data for Figs 1–3. (XLSX)

S2 Data Data for Figs A-C in S2 Text. (XLSX)

S3 Data Raw data and data dictionary. (XLSX)
