# Supplementary material for: Socioeconomic factors impact the risk of HIV acquisition in the township population of South Africa: A Bayesian analysis
Source: PLOS Glob Public Health. 2023 Jan 26;3(1):e0001502. doi: 10.1371/journal.pgph.0001502 (PMC10021863; doi:10.1371/journal.pgph.0001502)
Supplement: S1 Text — (PDF) [file pgph.0001502.s001.pdf]

Socioeconomic factors impact the risk of HIV acquisition in the township population of South Africa: a Bayesian Analysis

Supporting File 1. Posterior medians, 89% and 95% credible intervals (CrI) of unadjusted analyses

Table A. Posterior median, 89% and 95% credible intervals (CrI) of the unadjusted odds ratios of the impact of subdistrict of residence on new HIV infections

	Odds Ratio	89% CrI	95% CrI
**Township**			
Subdistrict B	1.71	1.30 - 2.26	1.22 - 2.39
Subdistrict C	1.36	1.02 - 1.79	0.97 - 1.91

Table B. Posterior median, 89% and 95% CrI of the unadjusted odds ratios of the impact of subdistrict of residence on having tested for HIV in the past six months

	Odds Ratio	89% CrI	95% CrI
**Township**			
Subdistrict B	0.74	0.64 - 0.86	0.62 - 0.89
Subdistrict C	1.36	1.18 - 1.56	1.14 - 1.61
