# Supplementary material for: Socioeconomic factors impact the risk of HIV acquisition in the township population of South Africa: A Bayesian analysis
Source: PLOS Glob Public Health. 2023 Jan 26;3(1):e0001502. doi: 10.1371/journal.pgph.0001502 (PMC10021863; doi:10.1371/journal.pgph.0001502)
Supplement: S2 Text — (PDF) [file pgph.0001502.s002.pdf]

Socioeconomic factors impact the risk of HIV acquisition in the township population of South Africa: a Bayesian Analysis

Supporting File 2. Bayesian logistic regression models stratified by sex.

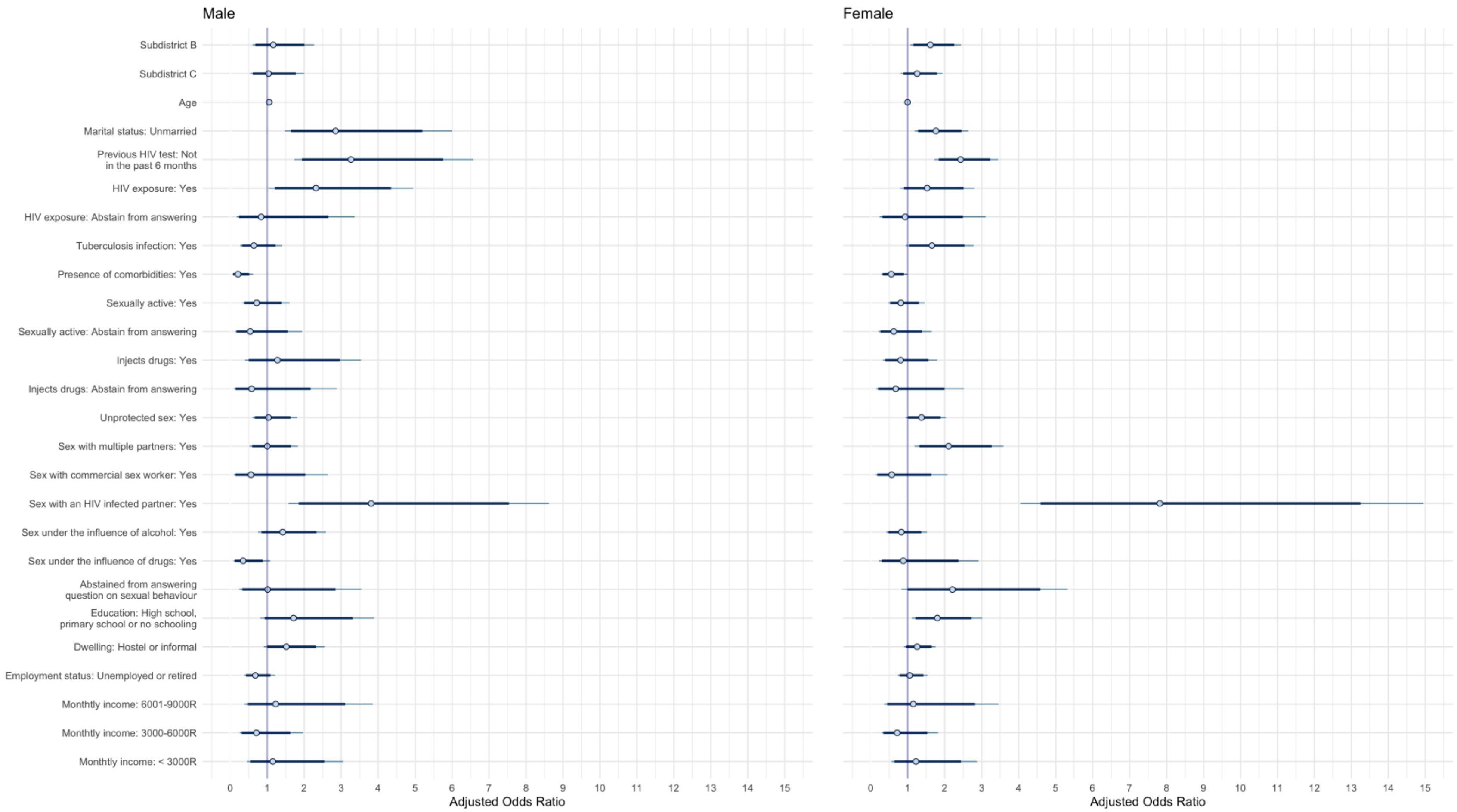

Figure A. Bayesian logistic regression model posterior intervals of adjusted odds ratios of HIV infection, stratified by sex. Points represent the posterior medians, thick segments represent the 89% credible intervals and thin segments represent the 95% intervals of the adjusted odds ratios.

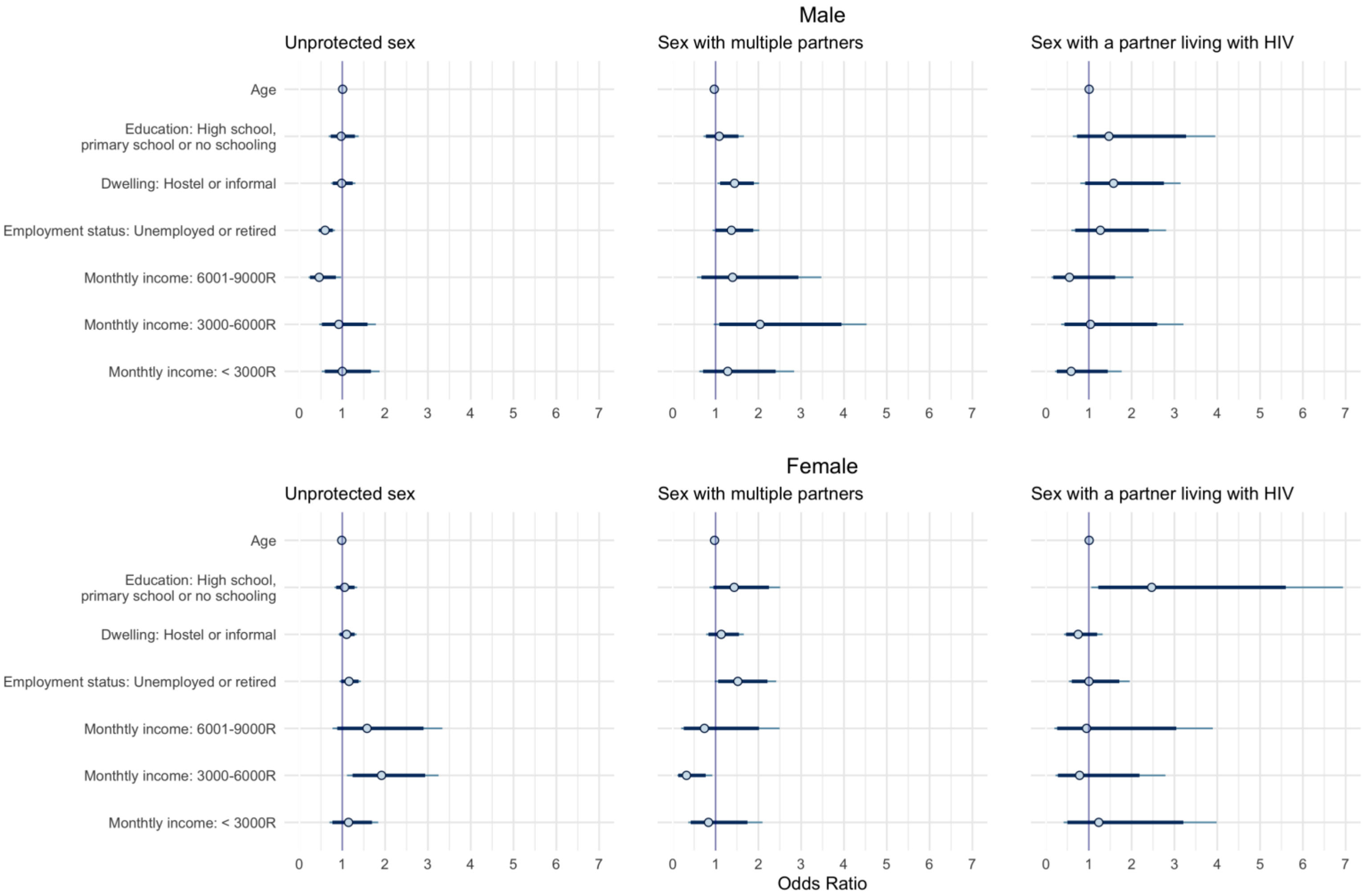

Figure B. Bayesian logistic regression model posterior intervals of adjusted odds ratios of the impact of socioeconomic factors on select behavioural factors, stratified by sex. Points represent the posterior medians, thick segments represent the 89% credible intervals and thin segments represent the 95% intervals of the adjusted odds ratios.

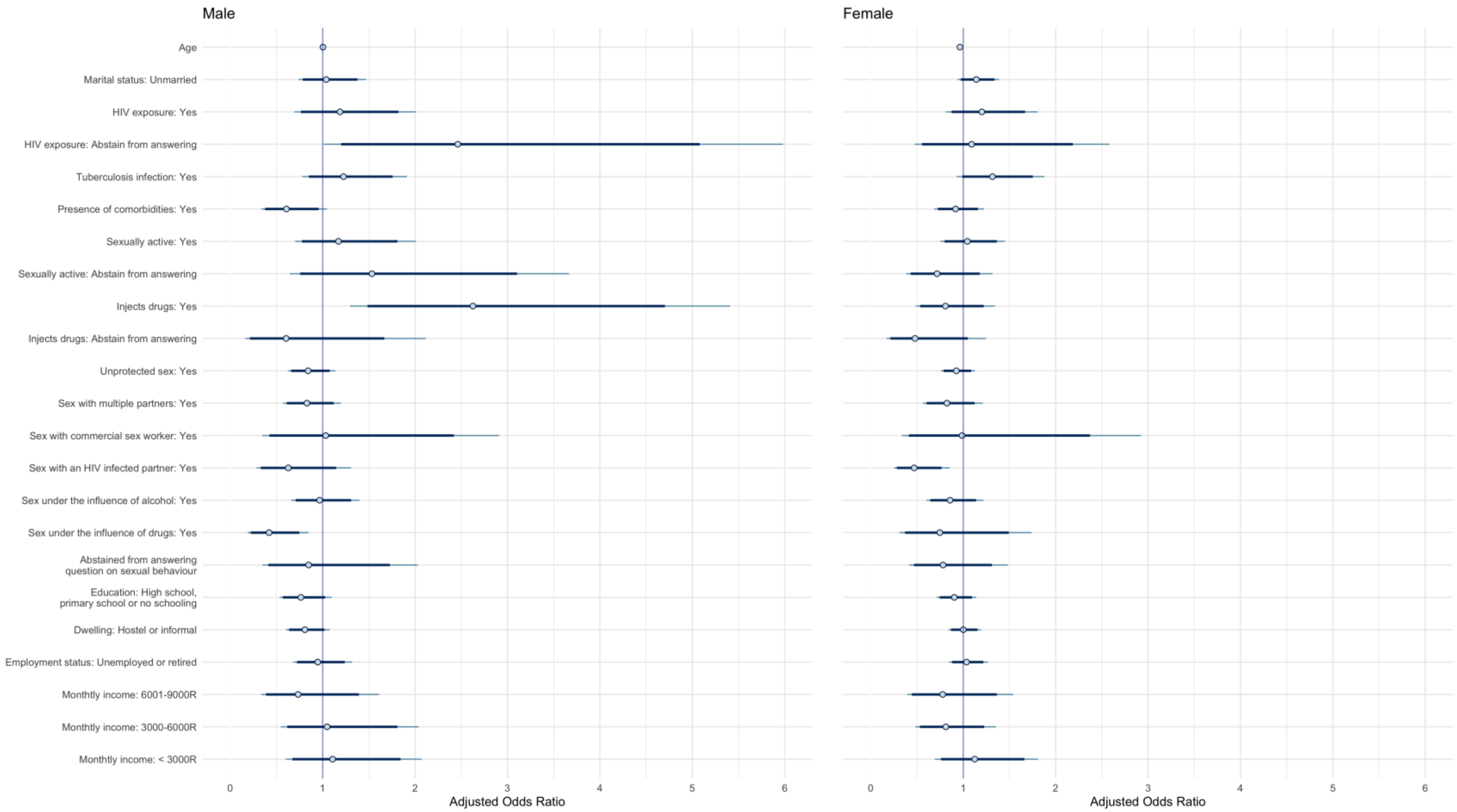

Figure C. Bayesian logistic regression model posterior intervals of adjusted odds ratios for having tested in the past six months, stratified by sex. Points represent the posterior medians, thick segments represent the 89% credible intervals and thin segments represent the 95% intervals of the adjusted odds ratios.
