# Supplementary material for: Socioeconomic factors impact the risk of HIV acquisition in the township population of South Africa: A Bayesian analysis
Source: PLOS Glob Public Health. 2023 Jan 26;3(1):e0001502. doi: 10.1371/journal.pgph.0001502 (PMC10021863; doi:10.1371/journal.pgph.0001502)
Supplement: S3 Text — (PDF) [file pgph.0001502.s003.pdf]

Socioeconomic factors impact the risk of HIV acquisition in the township population of South Africa: a Bayesian Analysis

Supporting File 3. Resampling model calibration plots

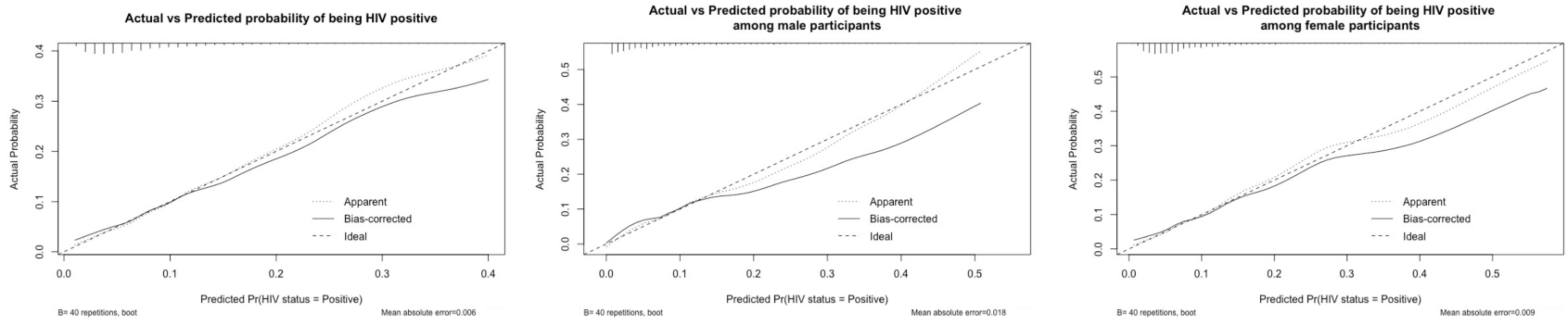

Figure A. Calibration curve with a bootstrap resampling validation for predicting HIV status. Validation was performed using 40 bootstrap samples. Dashed lines indicate the ideal model in which predicted and actual probabilities are perfectly identical. Dotted lines indicate the actual performance with apparent accuracy. Solid lines represent the bootstrap corrected estimate of the calibration curve. Histograms at the top represent the distribution of predicted values, giving an indication of where the majority of the data points are. More deviations from the ideal line are expected where there are fewer data points. The mean absolute error is the average absolute difference between the predicted and the actual probabilities; smaller values indicate better fit. The Cressie-van Houwelingen goodness-of-fit test p-values (larger values indicate no lack of fit) for the non-stratified model, the male participants model and the female participants model were 0.94, 0.82 and 0.80, respectively.

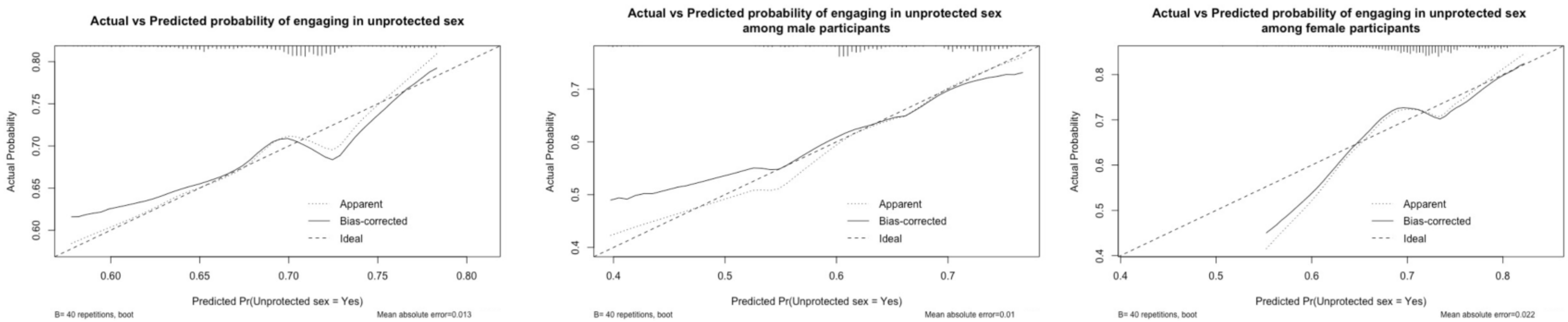

Figure B. Calibration curve with a bootstrap resampling validation for predicting unprotected sex. Validation was performed using 40 bootstrap samples. Dashed lines indicate the ideal model in which predicted and actual probabilities are perfectly identical. Dotted lines indicate the actual performance with apparent accuracy. Solid lines represent the bootstrap corrected estimate of the calibration curve. Histograms at the top represent the distribution of predicted values, giving an indication of where the majority of the data points are. More deviations from the ideal line are expected where there are fewer data points. The mean absolute error is the average absolute difference between the predicted and the actual probabilities; smaller values indicate better fit. The Cressie-van Houwelingen goodness-of-fit test p-values (larger values indicate no lack of fit) for the non-stratified model, the male participants model and the female participants model were 0.50, 0.92 and 0.16, respectively.

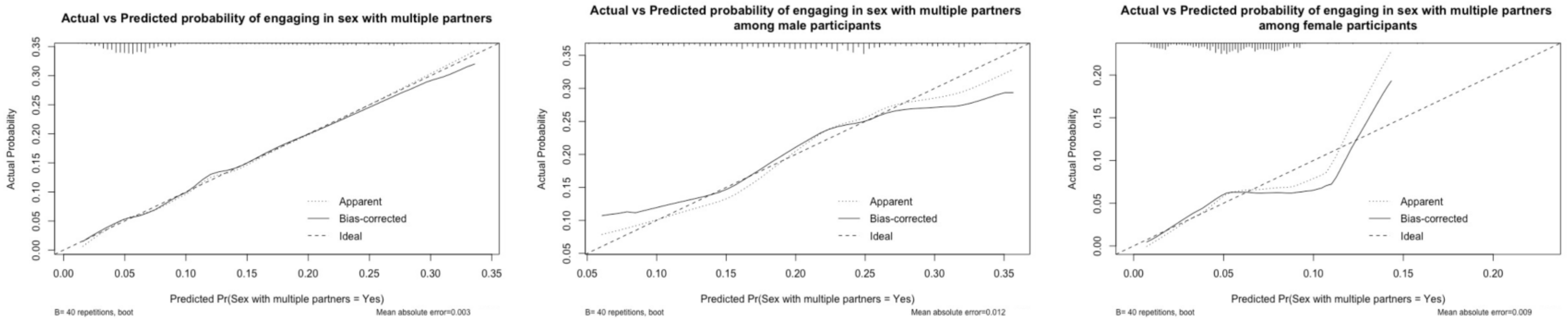

Figure C. Calibration curve with a bootstrap resampling validation for predicting sex with multiple partners. Validation was performed using 40 bootstrap samples. Dashed lines indicate the ideal model in which predicted and actual probabilities are perfectly identical. Dotted lines indicate the actual performance with apparent accuracy. Solid lines represent the bootstrap corrected estimate of the calibration curve. Histograms at the top represent the distribution of predicted values, giving an indication of where the majority of the data points are. More deviations from the ideal line are expected where there are fewer data points. The mean absolute error is the average absolute difference between the predicted and the actual probabilities; smaller values indicate better fit. The Cressie-van Houwelingen goodness-of-fit test p-values (larger values indicate no lack of fit) for the non-stratified model, the male participants model and the female participants model were 0.98, 0.78 and 0.78, respectively.

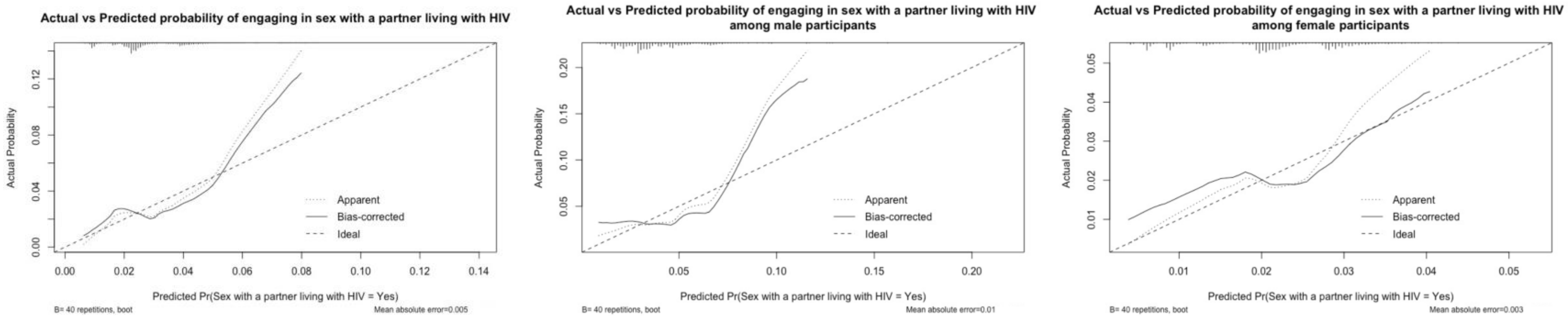

Figure D. Calibration curve with a bootstrap resampling validation for predicting sex with a partner living with HIV. Validation was performed using 40 bootstrap samples. Dashed lines indicate the ideal model in which predicted and actual probabilities are perfectly identical. Dotted lines indicate the actual performance with apparent accuracy. Solid lines represent the bootstrap corrected estimate of the calibration curve. Histograms at the top represent the distribution of predicted values, giving an indication of where the majority of the data points are. More deviations from the ideal line are expected where there are fewer data points. The mean absolute error is the average absolute difference between the predicted and the actual probabilities; smaller values indicate better fit. The Cressie-van Houwelingen goodness-of-fit test p-values (larger values indicate no lack of fit) for the non-stratified model, the male participants model and the female participants model were 0.57, 0.07 and 0.65, respectively.

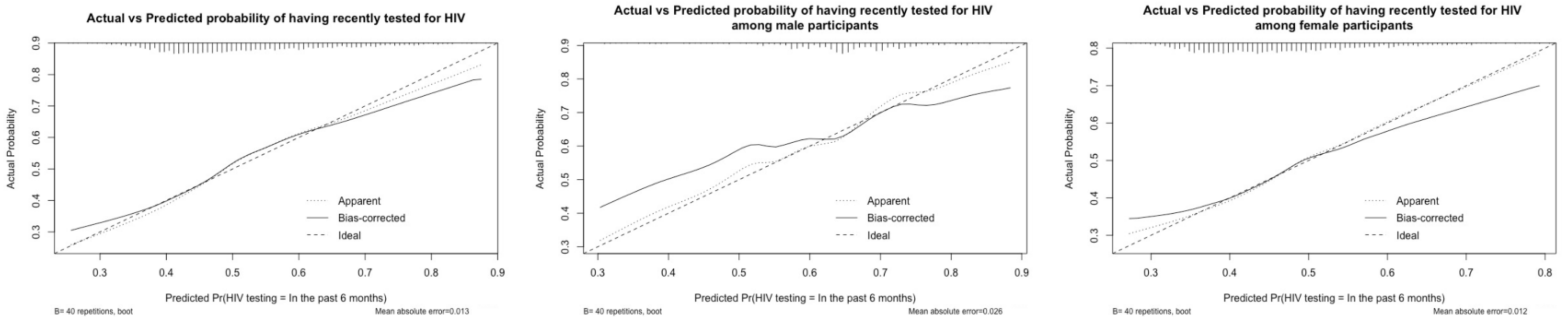

Figure E. Calibration curve with a bootstrap resampling validation for predicting HIV test in the past 6 months. Validation was performed using 40 bootstrap samples. Dashed lines indicate the ideal model in which predicted and actual probabilities are perfectly identical. Dotted lines indicate the actual performance with apparent accuracy. Solid lines represent the bootstrap corrected estimate of the calibration curve. Histograms at the top represent the distribution of predicted values, giving an indication of where the majority of the data points are. More deviations from the ideal line are expected where there are fewer data points. The mean absolute error is the average absolute difference between the predicted and the actual probabilities; smaller values indicate better fit. The Cressie-van Houwelingen goodness-of-fit test p-values (larger values indicate no lack of fit) for the non-stratified model, the male participants model and the female participants model were 0.46, 0.71 and 0.43, respectively.
